# Burnout Risk Profiles in Psychology Students: An Exploratory Study with Machine Learning

**DOI:** 10.3390/bs15040505

**Published:** 2025-04-09

**Authors:** M. Graça Pereira, Martim Santos, Renata Magalhães, Cláudia Rodrigues, Odete Araújo, Dalila Durães

**Affiliations:** 1 Psychology Research Centre (CIPsi), School of Psychology, University of Minho, 4710-057 Braga, Portugal; martimsantos@email.com; 2 Algoritmi Research Centre/LASI, University of Minho, 4800-058 Guimarães, Portugal; re_mag1419@hotmail.com (R.M.); dad@di.uminho.pt (D.D.); 3 Nursing Research Centre, University of Minho, 4710-057 Braga, Portugal; claudiailrodrigues15@gmail.com (C.R.); odete.araujo@ese.uminho.pt (O.A.); 4 School of Nursing, University of Minho, 4710-057 Braga, Portugal; 5 Health Sciences Research Unit: Nursing (UICISA: E), Nursing School of Coimbra (ESEnfC), 3045-043 Coimbra, Portugal

**Keywords:** burnout, psychological well-being, university students, risk profiles, healthy lifestyles, emotional regulation, psychological distress, machine learning

## Abstract

University students are at increased risk of developing burnout and psychological distress from high academic workloads and performance expectations. The purpose of this study is to analyze the relationship between psychological and lifestyle variables and academic burnout, as well as to identify burnout risk profiles in psychology students. This study used a cross-sectional design and included 274 Portuguese psychology students, the majority being undergraduates (72.6%). Participants were assessed on psychological well-being, psychological distress, difficulties in emotional regulation, type of diet, physical activity, sleep quality, and burnout. The results showed that psychological distress, difficulties in emotional regulation, and sleep quality were positively associated with burnout, while psychological well-being was negatively associated. Using machine learning algorithms, two distinct profiles were found: “Burnout Risk” and “No Risk”. A total of 62 participants were identified as belonging to the burnout risk profile, showing higher levels of distress, emotional regulation difficulties, poor psychological well-being and sleep quality, pro-inflammatory diet, and less physical activity. The accuracy of the three machine learning models—Random Forest, XGBoost, and Support Vector Machine—was 95.06%, 93.82%, and 97.53%, respectively. These results suggest the importance of health promotion within university settings, together with mental health strategies focused on adaptive psychological functioning, to prevent the risk of burnout.

## 1. Introduction

University students are at increased risk of developing burnout and psychological distress as a result of high academic workloads, performance expectations, and other external and internal demands related to the academic context (e.g., interpersonal relationships; Kaggwa et al., 2021; March-Amengual et al., 2022; Portoghese et al., 2018). Traditionally, burnout refers to “a state of mental and physical exhaustion caused by one’s professional life” (Freudenberger, 1974, p. 160). The development of the Maslach model helped to understand that burnout is a multidimensional construct that encompasses three major dimensions: (a) emotional exhaustion, (b) depersonalization (cynicism), and (c) lack of personal accomplishment (inefficacy; Maslach & Jackson, 1981; Maslach et al., 2001). Recently, burnout has been classified according to the context in which it occurs (e.g., parental burnout; Mikolajczak et al., 2020; Pereira et al., 2024) and differs from psychological distress (e.g., depression, stress) for this reason, being difficult to detect early on and prevented (Rössler, 2012). As such, Schaufeli et al. (2002) defined academic burnout as feelings of incompetence and exhaustion resulting from study demands, as well as a cynical and distant attitude toward academic pursuits.

In university students, burnout is characterized by feelings of exhaustion regarding the demands and the attitude towards studying, being negatively associated with academic and personal adaptation to university life (Özhan & Boyaci, 2022) and emotional dysregulation (Cooper et al., 2017). A recent systematic review and meta-analysis found that 24% of the students in this study showed clinically significant symptoms of depression, and 17% showed clinical symptoms of anxiety (Satinsky et al., 2021). The Portuguese study by Marôco et al. (2020) revealed that Biological Sciences, Exact Sciences, and Health Sciences students showed higher levels of burnout, with females presenting marginally higher burnout scores when compared to male students. Notwithstanding, Social Sciences students have been an underrepresented sample in most studies, particularly psychology students, although recent studies have found significant levels of burnout in this specific group of students (Laher et al., 2021; Martínez-Rubio et al., 2021; Paloș et al., 2019). The gap concerning psychology students highlights the need to develop exploratory studies focusing on these students from a biopsychosocial perspective, including the assessment of psychological variables and health behaviors for a more wide-ranging picture of the emergence of burnout in this group.

According to a recent systematic review, the prevalence of burnout in different dimensions, among university students, was estimated at 55.4% for emotional exhaustion, 31.6% for cynicism, and 30.9% for academic ineffectiveness, which indicates a moderate to high prevalence worldwide (Rosales-Ricardo et al., 2021). In addition, a systematic review and meta-analysis focusing on academic burnout among university students during the COVID-19 pandemic showed an increase in the global prevalence rates (41.8% to 56.3%), supporting burnout as a major public health concern (Abraham et al., 2024). Several sociodemographic, lifestyle, and psychological factors have been associated with higher levels of academic burnout in university students, such as being an upper-grade student, parents’ low education level (Liu et al., 2023), less physical activity, substance use (e.g., tobacco, alcohol; Liu et al., 2023; Nteveros et al., 2020), unhealthy diet (Nteveros et al., 2020), sleep problems (Allen et al., 2020; Arbabisarjou et al., 2015; Nteveros et al., 2020), and difficulties in emotional regulation (Iuga & David, 2024; L. Kong, 2024). Regarding gender, inconsistent and contradictory differences have been found between men and women in higher education contexts (e.g., Sabagh et al., 2018), although recent evidence suggests that male students have higher levels of academic burnout (Liu et al., 2023). Psychological distress has also been positively associated with academic burnout in several studies (e.g., Arbabisarjou et al., 2015; H. L. Chen et al., 2022; March-Amengual et al., 2022). Additionally, academic burnout has multifactorial consequences (e.g., health, psychological, physical, and occupational) and may exacerbate symptoms of psychological distress (e.g., depression) as well as predict suicidal ideation (Dyrbye et al., 2008), subjective academic performance, and dropout intention (Marôco et al., 2020). Thus, understanding and addressing academic risk factors for burnout and outcomes among university students, is imperative to inform the design of evidence-based intervention programs and be able to identify burnout vulnerability profiles, contributing to a decrease in dropout rates, promoting academic performance, and overall academic and psychological well-being. Students at high risk for burnout may benefit from targeted mental health interventions (Salmela-Aro & Upadyaya, 2014), highlighting the importance of educators and educational institutions’ consciousness to this issue and the need to act (e.g., referring to mental health professionals), since academic burnout is a significant predictor of worse academic achievement, as found in a meta-analysis of more than 100,000 students (Madigan & Curran, 2021).

It is well documented that academic burnout is negatively associated with psychological well-being among university students (A. U. Rehman et al., 2020; Ríos-Risquez et al., 2018; J. Yu & Chae, 2020). A previous longitudinal study found that emotional exhaustion plays a predictive role in psychological well-being, suggesting that this burnout dimension has a strong and significant adverse effect on students’ psychological well-being (Ríos-Risquez et al., 2018). Furthermore, recent research showed that adaptative emotional regulation strategies were negatively associated with academic burnout (Iuga & David, 2024) and positively associated with psychological well-being (Graça & Brandão, 2024), whereas difficulties in emotional regulation were linked to higher scores in all dimensions of academic burnout (i.e., emotional exhaustion, cynicism, and academic inefficacy; Iuga & David, 2024), and, in particular, expressive suppression (a component of emotional regulation) was negatively linked to students’ psychological well-being (Graça & Brandão, 2024). Also, L. Kong (2024) found that anxiety played a fully mediating role in the relationship between emotional regulation and academic burnout, which suggests that psychological functioning is the underlying pathway through which emotional regulation predicts burnout.

In adults, the practice of exercise, a good diet, and adequate sleep has been associated with less distress and more psychological well-being (Sanchez et al., 2019). A healthier lifestyle, including dietary patterns, physical activity, and good sleep quality, was associated with lower anxiety and depression (Bicalho et al., 2019), reduced stress levels (Santos et al., 2019), and less inflammation (Hart et al., 2021). An anti-inflammatory diet may prevent depressive and anxiety symptoms (Liu et al., 2023) and has been associated with better mental health (Guzek et al., 2021). Several studies showed a positive relationship between an inflammatory eating pattern and several chronic diseases, such as cancer (Shivappa et al., 2015), metabolic syndrome (Phillips et al., 2018), and depression (Bergmans & Malecki, 2017). Phillips et al. (2018), in a study with adults, found that a pro-inflammatory diet was associated with a higher risk of depressive and anxiety symptoms and a lower probability of better physical and mental well-being. Healthy nutrition and adequate sleep have also been associated with lower levels of stress and better performance (Di Renzo et al., 2021; Konjarski et al., 2018).

The promotion of physical activity has been considered a protective factor, associated with less stress, depression, employee absenteeism, and better performance and professional satisfaction (Proper & Van Oostrom, 2019). A recent study, conducted by Cao et al. (2022), identified physical inactivity, over 6 h per day, as a serious public health problem, contributing to the incidence of twelve cardiovascular, respiratory, autoimmune, neurological, and psychiatric pathologies.

Sleep also has a regulatory function, and its disturbance impacts the physical, mental, and social health of individuals. Excessive daytime sleepiness may result in sleep deprivation, obstructive sleep apnea, and other relevant clinical conditions, such as narcolepsy (Perlis et al., 2022). The alterations of the circadian rhythm are strongly associated with neuropsychiatric disorders, including cerebral lesions, bipolar disease, schizophrenia, Parkinson’s disease, and dementia (Meyer et al., 2022).

The literature is unanimous in recognizing the relevance and impact of healthy nutrition, physical exercise, and sleep quality in the prevention of physical and mental illnesses, impacting the individual’s personal, professional, and social life (Cao et al., 2022; Meyer et al., 2022). The practice of one or more health behaviors (sleep, diet, and exercise—the “Big Three”) is associated with better mental health and psychological well-being (Wickham et al., 2020).

Overall, research on psychological well-being and burnout among university students is crucial to understanding students’ needs. Identifying the factors that influence burnout may help higher education institutions implement effective interventions to improve the well-being of their students, professionals, and staff, and consequently, the quality of teaching. Hence, fostering psychological well-being among university students could be an important target to prevent and reduce academic burnout (A. U. Rehman et al., 2020). To promote students’ well-being, it is necessary to expand the knowledge within university settings, particularly regarding the relationship between students’ health behaviors (e.g., sleep quality, physical activity, and dietary pattern) and academic burnout, which remains unclear.

The current study was conceptually based on the job demand–resource model of burnout (Bakker & Demerouti, 2017; Demerouti et al., 2001). According to this theoretical model, there are two main types of workplace effects (i.e., demands and resources) related to the emergence of burnout. Demands encompass cognitive, emotional, and physical factors that lead to stress, exhaustion, and strain (e.g., difficulties in emotional regulation), while resources consist of a set of protective factors (e.g., physical exercise) that often help individuals cope adaptively with workplace tasks. The effects of resources and demands help to explain how individuals either counteract or exacerbate levels of burnout, respectively. Recently, Jagodics and Szabó (2022) proposed a new demand–resource model approach centered on burnout in university students, suggesting that the balance between demands and resources is key to preventing academic burnout. The job demand–resource model of burnout is a comprehensive framework useful to understand the phenomenon of burnout in multiple contexts, with growing applications to students’ academic settings (e.g., Hodge et al., 2019; Salmela-Aro & Upadyaya, 2014). The present study focused on psychology students, aimed: (1) to analyze the relationship between psychological (psychological well-being, psychological distress, and emotional regulation difficulties) and lifestyle (inflammatory diet, frequency of physical activity, and sleep quality) variables and academic burnout and (2) explore patterns in the data, enabling the identification of burnout risk profiles considering psychological and lifestyle factors, using machine learning algorithms. Traditional statistical methods have been widely used to explore the predictors of burnout; however, these methods often assume linear relationships between predictors and outcomes, which may not capture the complexity and non-linear interactions present in psychological and lifestyle data. Machine learning algorithms were chosen since the technique handles complex, high-dimensional datasets and can detect non-linear relationships between predictors.

The following hypotheses (H) were formulated: H1—greater psychological well-being, less psychological distress, fewer difficulties in emotional regulation, an anti-inflammatory diet, more sleep quality, and greater physical activity are expected to be associated with lower burnout; H2—unhealthy lifestyle patterns (poor sleep, pro-inflammatory diet, and less physical activity), lower psychological well-being, more psychological distress, and more emotional regulation difficulties are expected to configure a high-risk profile for academic burnout. We hope that the results of this study will inform future intervention programs aimed at preventing and reducing burnout and thereby promoting better academic and health outcomes.

## 2. Materials and Methods

### 2.1. Participants and Procedure

Psychology students were contacted through e-mail explaining the objective of this study, as well as an informed consent that ensured the confidentiality of the information and the voluntary nature of their participation. The inclusion criteria were: being 18 years of age or over; a psychology student in any study cycle (e.g., bachelor’s, master’s); and being enrolled at the university where the research was being conducted. The questionnaires were answered via Google Forms and took approximately 20 minutes. The study used a cross-sectional design, and the research project was approved by the Ethics Committee of a major public university in northern Portugal. Participants were 274 psychology students with a mean age of 22.05 (*SD* = 5.82), ranging from 18 to 49 years old. The vast majority of participants were female (89.4%) and undergraduate students (72.6%). Regarding their parents’ education level, most participants had both parents with completed secondary education (26.3 to 31.4%), and 24 participants (8.8%) reported having dependents under their care. One hundred and forty-three students (52.2%) attend higher education without the support of a scholarship, 173 (63.1%) returned home every day, 130 (47.4%) used public transports to get to the university, and a significant majority were placed in their first course choice (in this case psychology) (90.5%) and university choice (80.7%). Also, most participants have never failed an academic year (95.6%) and often studied alone (95.6%), at home (88.3%), and only 48 (17.52%) had working-student status. Finally, most participants were not involved in voluntary or associative movements (90.1%) or were involved in academic duties (89.8%). Table 1 shows the sociodemographic and clinical characteristics of the sample.

### 2.2. Instruments

#### 2.2.1. Sociodemographic and Clinical Questionnaire

This questionnaire was designed for this study and assessed sociodemographic (e.g., age, gender, course satisfaction) and clinical variables (e.g., use of psychoactive substances, general health status).

#### 2.2.2. Maslach Burnout Inventory—Student Survey (MBI-SS; Schaufeli et al., 2002; Portuguese Version by Marôco & Tecedeiro, 2009)

This self-report questionnaire consists of 15 items to assess burnout in students, divided into three subscales: emotional exhaustion, academic ineffectiveness, and cynicism. Each item is scored on a seven-point Likert scale, where 0 corresponds to “never” and 6 to “always”. The items corresponding to the academic inefficacy subscale (10 to 15) are inverted. Higher scores indicate higher levels of burnout. In the Portuguese version, Cronbach’s alpha varies from 0.79 (academic inefficacy) to 0.88 (cynicism). In this study, the alphas were 0.90 (emotional exhaustion and total scale), 0.92 (cynicism), and 0.85 (academic inefficacy).

#### 2.2.3. Psychological General Well-Being Index (QGBEP; Grossi et al., 2006; Portuguese Version by Barroso et al., 2018)

This self-report questionnaire consists of 6 items, where each item is answered on a five-point Likert scale in order to assess psychological well-being. The total scale scores range from 0 to 30. Higher scores indicate greater psychological well-being. In the Portuguese version, Cronbach’s alpha was 0.86, and in this study, it was 0.89.

#### 2.2.4. Depression, Anxiety, and Stress Scale (DASS21; Lovibond & Lovibond, 1995; Portuguese Version by Pais-Ribeiro et al., 2004)

This self-report instrument assesses anxiety, depression, and stress and includes 21 items, divided equally for each of the three sub-scales. Each item is answered on a four-point Likert scale, where zero indicates “did not apply to me at all”, and three means “applied to me very much or most of the time”, based on the last week. High scores indicate greater symptom severity. In the Portuguese version, Cronbach’s alpha for each subscale ranged between 0.50 (anxiety and stress) and 0.60 (depression). In this study, the alphas were 0.91 for the depression subscale, 0.87 for the anxiety subscale, 0.89 for the stress subscale, and 0.95 for the total scale.

#### 2.2.5. Difficulties in Emotion Regulation Scale (DERS; Gratz & Roemer, 2004; Portuguese Version by Coutinho et al., 2010)

This self-report instrument assesses the difficulties in emotional regulation. It consists of 36 items divided into six subscales: non-acceptance of emotional responses (6 items), difficulties in impulsive behavior (6 items), limited access to emotional regulation strategies (8 items), lack of emotional awareness (6 items), lack of emotional clarity (5 items), and inability to engage in goal-directed behavior (5 items) when experiencing negative emotions. Items are scored using a five-point Likert scale ranging from 1 “almost never” to 5 “almost always”. Higher scores indicate higher levels of emotional regulation difficulties. In the Portuguese version, Cronbach’s alpha was 0.92 for the total scale, and in this study, it was 0.94.

#### 2.2.6. International Physical Activity Questionnaire—Short Version (IPAQ; Craig et al., 2003; Portuguese Version by Campaniço, 2016)

This questionnaire is a self-report instrument comprising 7 items that assess levels of physical activity over the last seven days, allowing individuals to be classified as vigorously active, moderately active, and insufficiently active/inactive. Part (a) of each item aims to identify how many days a week physical activity is performed (vigorous, moderate, or walking) and is scored on an eight-point Likert scale, where 0 corresponds to “no days” and 7 to “every day”; and part (b) corresponds to the time, in minutes, dedicated to this physical activity. Higher scores indicate greater physical activity.

#### 2.2.7. Pittsburgh Sleep Quality Index (PSQI; Buysee et al., 1989; Portuguese version by Del Rio João et al., 2017)

This instrument aims to assess sleep quality over the last month and consists of 19 items, categorized into seven specific components: (1) subjective sleep quality, (2) sleep latency, (3) sleep duration, (4) sleep efficiency, (5) sleep disturbances, (6) use of sleep medication, and (7) daytime dysfunction. The sum of these components results in an overall score (sleep quality) ranging from 0 to 21. The cutoff point for the total scale is 5, suggesting that scores higher than this indicate a poorer quality of sleep.

#### 2.2.8. Food Frequency Questionnaire (QFA; Willett, 1998; Portuguese Version by C. M. d. M. Lopes, 2000; C. Lopes et al., 2007)

This instrument identifies dietary patterns and consists of 86 items that assess the frequency of consumption of certain foods and drinks over the last 12 months. Each item is scored on a nine-point Likert scale, where 0 corresponds to “never or less than once a month” and 8 to “six or more per day”. To obtain a food consumption score, the frequency reported for each item needs to be multiplied by the respective standard average portion, in grams (g), and by a seasonal variation factor for foods consumed in specific seasons (0.25 was considered the average seasonality factor for three months). The foods were converted into nutrients by the Public Health Institute of the University of Porto, in Portugal, using the Food Processor Plus software (ESHA Research, Salem, Oregon) and based on foods’ nutritional information, according to the reference data developed by the Nutritional Epidemiology Unit of the Faculty of Medicine of the University of Porto (Portugal). After this process, the dietary inflammatory potential was calculated using the dietary inflammatory index (DII; Shivappa et al., 2013). The effect of up to 45 dietary parameters was considered and scored with +1 (for pro-inflammatory parameters), −1 (for anti-inflammatory parameters), and 0 (for parameters with no effect on inflammatory markers). In this study, the DII was calculated based on 28 parameters (i.e., alcohol, vitamin B12, vitamin B6, caffeine, carbohydrates, cholesterol, calories, total fat, fiber, folate, iron, magnesium, monosaturated fat, niacin, omega3, omega6, protein, polyunsaturated fat, riboflavin, saturated fat, selenium, thiamine, trans fat, retinol [vitamin A], vitamin C, vitamin D, vitamin E, and zinc). DII scores can range from +7.98 (strongly pro-inflammatory) to −8.87 (strongly anti-inflammatory). Positive scores indicate a pro-inflammatory diet.

### 2.3. Data Analysis

The dataset comprised a total of 274 entries and a total of 40 columns, including an identification number, sociodemographic variables (e.g., age, gender, and course satisfaction), and clinical variables (e.g., health status, tobacco use, substance use, alcohol consumption, and psychological support) as well as psychological (difficulties in emotional regulation, psychological distress, psychological well-being, and academic burnout) and lifestyle (sleep quality, dietary inflammatory index, physical activity) variables. To test the relationships between the psychological and lifestyle variables and academic burnout, the Pearson correlation coefficient was used. Also, descriptive statistics were performed to describe the sociodemographic and clinical characteristics of the sample, using IBM SPSS Statistics (Statistical Package for the Social Sciences) version 29. In addition, an exploratory analysis was conducted to analyze the distribution within every variable, identifying relevant variables for cluster modeling, and to gain insights into the quality of the data as well as to identify interesting patterns that could generate hypotheses about hidden information.

An explanatory burnout model was created using the analytical methodology of Structural Equation Modeling (SEM) with the lavaan package in R to assess the relevance of the different predictors of burnout. Several models were tested, including both sociodemographic and health-related variables, but the final model consisted of the following variables: psychological well-being, psychological distress, emotional regulation, physical activity, sleep quality, and the inflammatory index. This model showed good fit indices (CFI: 0.956, TLI: 0.917, RMSEA: 0.068, SRMR: 0.038), and the variables were subsequently used for the prediction of burnout risk profiles. For burnout risk prediction, supervised machine learning algorithms—XGBoost, Random Forest, and Support Vector Machines (SVM)—were applied. Unlike clustering techniques (e.g., k-means), where group data are based on inherent patterns without predefined outcomes, supervised learning was chosen because our goal was to classify individuals into “at-risk” and “no-risk” categories based on the predictors identified through SEM. The data was split into 70% for training and 30% for testing. To prevent overfitting and ensure model generalizability, 10-fold cross-validation was implemented, where the data were divided into 10 subsets, and the models were trained on different combinations of these subsets. The models were evaluated using precision, accuracy, recall, and F1-score.

## 3. Results

### 3.1. Relationship Between Psychological and Lifestyle Variables with Academic Burnout

The results showed a positive association between psychological distress (*r* = 0.560, *p* < 0.01), difficulties in emotional regulation (*r* = 0.457, *p* < 0.01), sleep quality (*r* = 0.336, *p* < 0.01), and burnout. In turn, psychological well-being (*r* = −0.610, *p* < 0.01) was negatively associated with burnout. No significant associations were found between physical activity, dietary inflammatory index, and academic burnout (Table 2).

### 3.2. Cluster Analysis

To identify risk profiles, cluster analyses were carried out, and participants were grouped according to the previously defined relevant variables included in the theoretical model of structural equations: psychological distress (DASS21), psychological well-being (QGBEP), difficulties in emotional regulation (DERS), sleep quality (PSQI), physical activity (IPAQ), and inflammatory diet (DII).

The dataset was well structured from the start, so the pre-processing steps were simple, including removing null and invalid values. In cases of unanswered questions (only seven throughout the entire dataset), the missing value was replaced by the mean score for that specific variable. The data were normalized to ensure that each variable had the same influence on the formation of the clusters. The k-means method was used to segment the dataset into two distinct burnout risk groups: risk of burnout and no risk of burnout.

The average of each variable was calculated for each cluster to understand the specific characteristics of each group. Minimum, maximum, and mean values were also calculated for the variables in each cluster, providing an overview of the distributions in each risk profile and detailing the amplitude and average value for each variable in each group.

**Figure 1:**
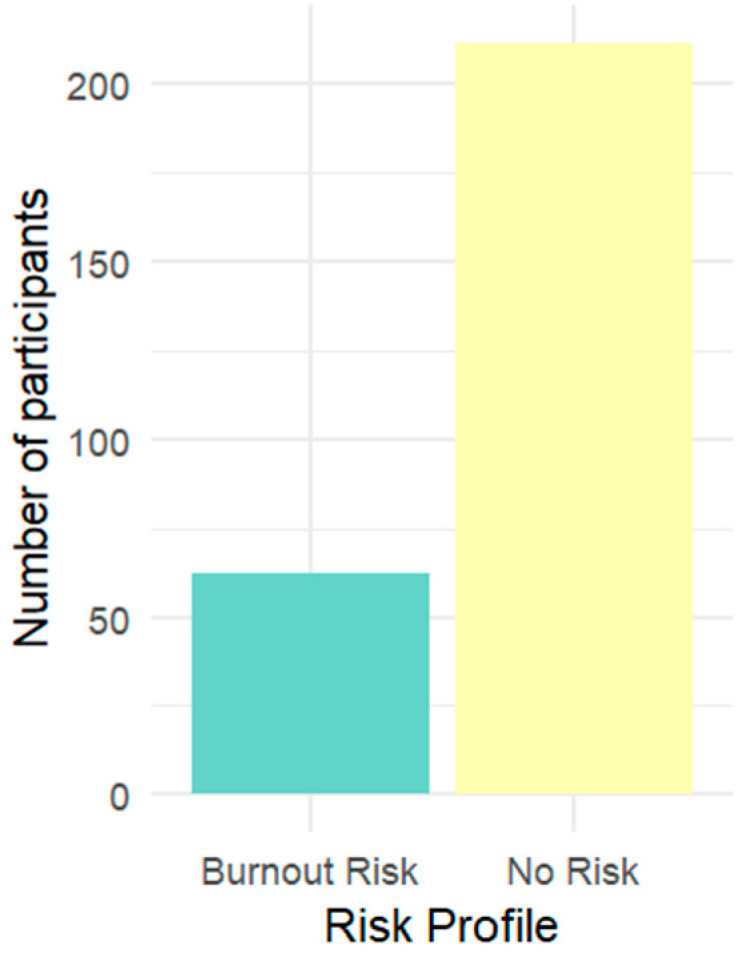
Risk cluster distribution.

The final data, including the clusters/risk profile, were saved in the dataset for future analysis. Figure 1 shows the distribution per risk cluster. A total of 62 participants were identified as being at “Burnout Risk”, while the remaining 211 were categorized as “No Risk”. This profiling is helpful in better understanding the characteristics of individuals who are at risk of burnout and those who are not, as well as signaling, in a timely manner, those who might be at risk of burnout, allowing for timely intervention. Table 3 shows the mean scores for each of the considered variables in the risk profiling.

In the no-risk profile, the mean values of psychological distress, emotional regulation, and sleep quality were significantly lower, indicating less distress, more emotional regulation, and better sleep quality. Physical activity was slightly higher, as well as a healthier diet (a negative score indicates an anti-inflammatory diet). All of these are in line with a more balanced and healthier lifestyle.

The at-risk profile presented the highest scores on distress, emotional regulation difficulties, and poor sleep quality. Additionally, although with a small difference, physical activity scores were slightly lower, suggesting that individuals targeted as being at risk of burnout exercise less. Regarding diet habits, the mean score was 1.69, which indicates a less healthy diet. All these characteristics were present in participants who have been flagged as being at risk of burnout, suggesting that poor well-being, high psychological distress, less emotional regulation, poor sleep quality, little physical exercise, and a pro-inflammatory diet are linked to burnout. These mean scores were fundamental in defining risk levels and suggest that factors such as mental health, emotional regulation, sleep quality, and lifestyle play a central role in determining the risk of burnout among the participants.

The cutoff values reported for each variable within the identified risk profiles were calculated by summarizing the minimum and maximum values of each variable for the participants assigned to each cluster. The clustering algorithm, in this case K-means, grouped individuals based on their similarity across the selected variables without explicitly establishing boundaries for the clusters. These cutoffs represent the range of values observed within each cluster, and provide descriptive insights into the profiles (Table 4).

The cutoff scores reveal distinct characteristics of the two identified risk profiles. These findings contrast profiles on psychological, behavioral, and lifestyle factors between the two groups.

### 3.3. Prediction of Burnout Risk Profiles

The burnout risk profiles were predicted using three supervised machine learning algorithms: Random Forest, XGBoost, and SVM (Support Vector Machine). These algorithms were chosen over traditional statistical approaches such as logistic regression or discriminant analysis due to their ability to handle complex, non-linear relationships and interactions between predictors, which are often difficult to capture with linear models. In fact, supervised ML algorithms are particularly useful when the relationships between the variables are not strictly linear, which is often the case in psychological and lifestyle data, like the one used in this study. Thus, the independent variables (predictors) and dependent variables (variable to be predicted) are defined, following the data used in the SEM model and the cluster analysis. The dependent variables were the total variables of well-being, psychological distress, emotional regulation, sleep quality, physical exercise, and inflammatory index.

The data were divided into 70% for training and 30% for testing. A 70–30 split helps to better detect overfitting, ensure a robust number of training data, and more rigorously assess the model’s ability to generalize, an important concern in multi-class predictions such as burnout risk profiles. Since the procedure entails a classification problem, i.e., there are classes or categories to be predicted, such as burnout risk profiles, the metrics used to evaluate the models were precision, accuracy, recall, and F1-score (Table 5).

Random Forest showed strong accuracy and balanced precision and recall, but it slightly underperformed compared to XGBoost. SVM demonstrated high precision but had lower recall, indicating it excelled in specific cases but missed some positives. XG Boost showed the best performance in accurately predicting the burnout risk profiles. This was particularly effective in minimizing false positives and negatives and accurately identifying individuals at high risk of burnout.

Overall, the variables of psychological well-being, psychological distress, emotional regulation, sleep quality, physical activity, and inflammatory index, with respect to XGBoost, showed great model performance in accurately predicting burnout risk profiles.

## 4. Discussion

This study analyzed the influence of psychological and lifestyle factors on academic burnout and established two distinct burnout risk profiles among psychology students. The results of the present study showed that psychological distress, difficulties in emotional regulation, sleep quality, and psychological well-being were the factors most strongly associated with academic burnout in psychology students, which is aligned with the job demand–resource model of burnout (Bakker & Demerouti, 2017; Demerouti et al., 2001). Grounded in this theoretical framework, the main findings of the current study suggest that psychology students who face high demands (e.g., psychological distress, and difficulties in emotional regulation) and report a lack of resources/protective factors (e.g., fewer health behaviors) may be at risk of developing significant levels of academic burnout.

The final SEM model revealed that psychological well-being emerged as a particularly strong predictor, reinforcing prior evidence that persistent stress and negative emotional states significantly contribute to the exacerbation of university students’ burnout levels (Yusoff et al., 2021). In line with this evidence, recent studies have shown a robust correlation between psychological distress and burnout (H. L. Chen et al., 2022; Emerson et al., 2023). Chronic exposure to psychological stressors depletes emotional resources, thereby increasing vulnerability to burnout (Demerouti et al., 2001). While previous research has primarily focused on medical students (Yusoff et al., 2021), the mechanisms underlying the relationship between psychological distress and burnout are broadly applicable to psychology students, who frequently face substantial academic demands. Therefore, interventions aimed at reducing unnecessary psychological pressures and avoidable stressors, which do not contribute to academic success, could serve as a protective strategy to mitigate the risk of burnout.

The results of the present study indicated that difficulties in emotional regulation represented both a risk factor and a predictor of academic burnout. These findings are consistent with the previous literature in which maladaptive emotional regulation strategies, such as avoidance, emotional suppression, and venting, were associated with intensified emotional exhaustion and cynicism (X. Yu et al., 2022). A recent meta-analysis further supports this evidence, showing that children and young people with difficulties in emotional regulation are more likely to experience symptoms of burnout, including emotional exhaustion, cynicism, and lack of efficiency (Iuga & David, 2024). Similarly, Chalikkandy et al. (2022) found that university students with better emotional regulation skills reported lower levels of burnout. Consistent with this outcome, previous research has shown that maladaptive cognitive and emotional coping strategies increase burnout risk in university students (Vizoso et al., 2019). These findings suggest that students with poor emotional regulation are more vulnerable to academic stress as they struggle to manage stressors effectively (X. Yu et al., 2022), amplifying, as a result, the risk of burnout. A recent study also suggests that daily work-related emotional exhaustion may be effectively managed through cognitive reappraisal of emotions and a reduced reliance on emotional suppression, highlighting the importance of emotional and environmental support to prevent burnout (Lim et al., 2024). Given this evidence, promoting a healthy university environment and training in emotional regulation (e.g., heart-focused breathing) could be key preventive measures against burnout. Therefore, evidence-based interventions, such as mindfulness-based approaches (da Silva et al., 2023), and brief, transdiagnostic cognitive-behavioral (group) interventions (Finch et al., 2024) showed high efficacy in reducing psychological distress symptoms and promoting psychological well-being in university students. In addition, psychoeducational programs (e.g., emotional intelligence) may help proactively prevent maladaptive psychological outcomes within university settings (Seppälä et al., 2020).

Several studies have identified a negative association between academic burnout and psychological well-being in university students (A. U. Rehman et al., 2020), particularly in medical students (J. Yu & Chae, 2020). This evidence corroborates the findings of the present study, in which lower psychological well-being was associated with higher burnout levels. Interestingly, a study involving nursing students concluded that emotional exhaustion negatively predicted psychological well-being (Ríos-Risquez et al., 2018), which contrasts with our results, in which psychological well-being emerged as a negative predictor of burnout. Taken together, these results highlight a potential bidirectional relationship between psychological well-being and burnout, underscoring the need for longitudinal studies to explore the causal direction and mediating variables in this relationship. Future research on university students should investigate whether improving psychological well-being may operate as a protective factor against academic burnout or if reducing burnout will result in enhanced psychological well-being.

Sleep quality was identified as another significant contributor to academic burnout among psychology students, which underlines the importance of addressing sleep hygiene in the prevention of burnout in academic environments. This finding is consistent with the literature, which has confirmed that poorer sleep quality increases levels of emotional exhaustion (Allen et al., 2020; Amaral et al., 2021; Hakimi et al., 2024; L. N. Kong et al., 2023), cynicism (Allen et al., 2020; Hakimi et al., 2024; L. N. Kong et al., 2023), and lack of efficiency (Allen et al., 2020). The association between sleep and burnout may be explained by the critical role that sleep plays in emotion regulation and cognitive functioning, both of which are essential for managing academic stress. A prior study has shown that inadequate sleep was associated with exacerbated psychological distress and impaired cognitive functions (e.g., attention and memory), which are crucial for managing academic tasks successfully. Disruption of these cognitive processes may hinder students’ ability to manage academic demands, potentially leading to higher levels of stress and increasing the risk of academic burnout (Amaral et al., 2021). Moreover, such outcomes corroborate the findings of Naderi et al. (2021), suggesting that sleep quality may be a predictor of academic burnout in undergraduate nursing students. Despite this, a study focused on medical students by Pagnin et al. (2014) concluded that academic burnout and sleep problems showed relevant bidirectional effects, particularly in the first academic years, which underlines the need for further longitudinal studies to clarify these potential mutual influences.

The results of this study further indicate that physical activity was not significantly associated with academic burnout. This outcome differs from previous evidence, showing a negative association between physical activity and burnout among medical and nursing students, respectively (S. Rehman et al., 2024; Taylor et al., 2022). The mixed results could be explained by differences regarding the sample’s characteristics and size, emphasizing the need for future research to employ bigger samples and to focus on university students from different professional and academic backgrounds. Interestingly, in the present study, physical activity was identified as a predictor of academic burnout, which is in line with recent literature (A. R. Lopes & Nihei, 2020; S. Rehman et al., 2024), suggesting that students engaging in regular physical exercise are at a lower risk of developing academic burnout (A. R. Lopes & Nihei, 2020). Physical activity predicted burnout, but no significant correlation was found between this variable and burnout could probably be explained by the differences between the types of analysis used. Correlation analysis typically measures linear associations, and physical activity may have a threshold effect (non-linear effect) on academic burnout, given the complexity of this construct. For example, only a specific level of physical activity (e.g., sedentary) may have an impact on burnout beyond a certain point, which cannot be detected in a correlation analysis. Future studies with a longitudinal design are recommended to determine causal relationships between physical activity and academic burnout and examine the impact of differences in physical activity levels (e.g., moderate and vigorous) on burnout.

Similar to physical activity, the results also showed that the DII did not correlate significantly with academic burnout but emerged as a predictor, suggesting that the relationship between a pro-inflammatory diet and burnout may be more intricate than initially expected. A recent study found a negative correlation between dietary habits and the dimensions of burnout in students from a private university (Estrada Araoz, 2024). Despite that, to the best of our knowledge, there is a knowledge gap regarding the relationship between the DII and academic burnout. Previous research showed that a more pro-inflammatory diet has been linked to an increased risk of adverse mental health outcomes, including poorer psychological well-being, depressive symptoms (Phillips et al., 2018), and overall psychological distress (G.-Q. Chen et al., 2021). Moreover, G.-Q. Chen et al. (2021) argued that psychological distress contributes to an increased consumption of high-energy and high-fat foods, thereby resulting in a higher DII score. These findings suggest that the relationship between a pro-inflammatory diet and psychological distress, which shares similar pathways with burnout, is potentially bidirectional. The lack of a significant correlation may suggest that inflammation does not have a simple linear relationship with academic burnout; however, a pro-inflammatory diet probably exerts its influence through more indirect or long-term mechanisms (e.g., chronic stress; Rohleder, 2019) on burnout. In addition, the predictive role of an inflammatory diet may reflect the importance of chronic inflammatory processes in predicting the risk of burnout. Thus, cohort and longitudinal studies are needed to further elucidate the relationship between the DII and academic burnout, including other subgroups of university students, and to explore the causal mechanisms linking dietary inflammation to burnout.

Furthermore, the SEM model and the clustering algorithm provided cutoff points for each major predictor, which, from a clinical perspective, is critical for the early identification of students at risk of burnout and for describing the characteristics associated with each risk profile. Psychology students, in the burnout risk group, reported higher psychological distress scores (24.6), indicating higher levels of anxiety, depression, and stress. The higher distress not only reflects the severity of the symptoms, but also points to an impaired ability to manage the emotional demands of academic life, which, in turn, may increase the risk of burnout. Indeed, a study involving 1174 university students from 53 Portuguese higher education institutions reported that 46% of participants exhibited depressive symptoms, 39% experienced symptoms of anxiety, and 21% showed symptoms of stress (Silva et al., 2024). The latter findings corroborate the present results, reinforcing the prevalence of mental health challenges among university psychology students and the urgent need for targeted interventions. The group of students at risk of burnout also showed significant difficulties in emotional regulation and lower psychological well-being, with average values of 96.6 and 12.9, respectively. University students face several academic stressors daily, and those who resort to maladaptive emotional and cognitive strategies are more likely to experience higher emotional exhaustion, increased cynicism, and a diminished sense of accomplishment (Vizoso et al., 2019; X. Yu et al., 2022). These maladaptive strategies are also connected to reduced psychological well-being (Ponce & Caguana Telenchana, 2023), further emphasizing the critical role of emotional regulation in mitigating burnout risk.

Regarding sleep quality, students at risk of burnout exhibited higher PSQI scores (8.10), indicating significantly poorer sleep quality compared to those not at risk. This sleep quality may intensify emotional and cognitive difficulties, thereby contributing to burnout risk. Additionally, physical activity scores were slightly lower in this group of students (1.79), suggesting a tendency to engage in less physical activity. This finding is consistent with previous research, which underscores the negative impact of sedentary behavior on mental health, particularly among university students (Verhavert et al., 2020). Furthermore, evidence also supports the positive effects of physical exercise in mitigating burnout symptoms (Rosales-Ricardo & Ferreira, 2022). These findings highlight the potential benefits of promoting physical activity as part of the burnout prevention strategy. Consequently, they also underline the need for further research to better understand how exercise may influence burnout outcomes by exploring the potential mediating factors between physical activity and burnout risk.

Finally, regarding the DII, students identified as at risk presented higher scores (1.69), which indicates a pro-inflammatory diet. There is a well-established consensus in the literature regarding the association between an inflammatory diet and mental health problems (G.-Q. Chen et al., 2021; Selvaraj et al., 2022). Specifically, a recent study found that individuals following a pro-inflammatory diet and engaging in physical inactivity face a greater risk of depressive symptoms compared to those adhering to an anti-inflammatory diet and maintaining regular physical activity (Wang et al., 2023). As previously discussed, this is the first study to explore the relationship between the DII and academic burnout.

Overall, the group of students not at risk of burnout showed lower cutoff points and scores indicative of better emotional regulation (79.6), lower distress (14.7), greater psychological well-being (17.6), and a healthier lifestyle. This distinct profile suggests that maintaining emotional regulation and psychological well-being is associated with a reduced risk of burnout. Thus, the lifestyle observed in this group emphasized the importance of healthier lifestyle choices in preventing academic burnout. These insights also highlight the importance of adopting holistic approaches to mental health and the critical need for multifaceted interventions that prioritize healthier lifestyle behaviors, including improved sleep quality, regular physical activity, and adherence to an anti-inflammatory diet.

The predictive model, employing the XGboost machine learning algorithm, was able to categorize students into two distinct profiles with 97.53% accuracy and 90.00% precision. These results highlight the robustness of the model and, consequently, the findings of the present study. The use of supervised machine learning algorithms offered several advantages over traditional methods like logistic regression or discriminant analysis, as simpler models could have identified basic associations between variables, while machine learning allows the detection of complex, non-linear relationships and interactions between psychological and lifestyle data such as psychological distress, emotional regulation, and sleep quality. The XGBoost algorithm, with its high predictive accuracy and ability to minimize false positives and negatives, provided a more reliable classification of students into burnout risk profiles, which would have been challenging to achieve using traditional models. These models also allowed us to identify combinations of predictors that jointly contributed to higher burnout risk, providing a more nuanced understanding of how multiple factors interact to influence academic burnout. For example, the combination of emotional regulation difficulties with poor sleep quality was found to significantly predict burnout risk, an interaction that might not have been detected in simpler models.

The present study has several strengths, such as the inclusion of lifestyle factors, with a particular emphasis on the dietary inflammatory index. Additionally, machine learning techniques led to a predictive model for academic burnout risk in psychology students which holds significant value to higher education institutions, particularly the screening of university students and, thereby, the timely identification of those requiring preventive interventions to mitigate the risk of burnout. However, this study also presents some limitations that should be acknowledged, the first of which is that the findings should be interpreted with caution due to the small sample size, consisting solely of psychology students from a single major university in Northern Portugal. In addition, this exploratory study used a cross-sectional design, which limits the establishment of causal relationships. In this line, another related limitation is the potential for reverse causation, since the observed relationship may reflect an inverse causal direction rather than the hypothesized effect. For example, there is evidence of a reciprocal relationship between difficulties in emotional regulation and burnout levels (Lim & Lee, 2025), and this cyclical relationship could not be effectively captured in a cross-sectional framework. Furthermore, given the potentially dynamic and complex nature of burnout, the absence of a longitudinal dimension does not allow for a direct and conclusive assessment of the temporal relationships between the constructs under study (e.g., Lim et al., 2025). Therefore, future studies with a longitudinal design should include larger and more representative samples of psychology students. Finally, to gain a comprehensive understanding of academic burnout risk among university students, further research should include students from other professional areas and explore gender as a predictor or moderator in diverse and larger samples.

### Implications for Practice

Although previous studies have highlighted medical, nursing, engineering, information technology (Rosales-Ricardo et al., 2021), biological sciences, and exact sciences students as those at the highest risk for academic burnout (Marôco & Assunção, 2020), the present study revealed that psychology students, despite being underrepresented in the literature, also exhibit significant vulnerability. The results of the present study also provided cutoff points for earlier screening of academic burnout risk. From an institutional perspective, our findings are also important for raising awareness within academia regarding the vulnerability of psychology students and emphasizing the need for preventive intervention plans aimed at promoting mental health in the academic context.

The findings also suggest that strategies focused on mental health literacy, particularly in emotional regulation skills, could positively influence the psychological well-being of university students and mitigate the risk of academic burnout. According to a recent systematic review focused on the impact of mental health literacy training programs on university students, this type of preventive intervention has the potential to mitigate the effects of recognized risk factors and help promote students’ overall well-being, contributing, for instance, to enhance (adequate) help-seeking behavior (Reis et al., 2021). Therefore, nutritional and health literacy, with a focus on the benefits of an anti-inflammatory diet, sleep quality, and regular physical activity, represent emerging areas of attention that could be beneficial and integrated into the intervention programs offered to students. Overall, higher education institutions should adopt a stepped-care approach to optimize access to mental health care and responsiveness, thereby enabling the transition to an active preventive paradigm. The stepped-care approach has been referenced as a good practice for improving mental health outcomes in university settings (Gago et al., 2023). Preliminary findings from a major Portuguese university suggested that the implementation of such a model would allow an improvement in the quality and efficacy of mental health services, through the early identification of needs, tailoring interventions to specific needs, and providing mental health initiatives according to the different levels of students’ needs (Marques et al., 2024).

## 5. Conclusions

Based on the results presented in this study, the machine learning models used (Random Forest, XGBoost, and SVM) showed high accuracy in classifying students into burnout risk profiles, with XGBoost standing out as having the best overall performance. These results indicate that factors such as psychological well-being, difficulties in emotional regulation, sleep quality, physical activity, and an inflammatory diet play a significant role in determining the risk of academic burnout. The models reinforce the relevance of addressing these factors in the university context, suggesting that interventions aimed at promoting mental health and healthy lifestyles can be effective in reducing the risk of burnout. Furthermore, the use of machine learning algorithms to identify risk profiles proved to be a powerful tool for early detection and for targeting preventive strategies. Therefore, the application of machine learning models not only contributed to a deeper understanding of the factors associated with burnout but also offered practical means for personalized interventions, promoting better academic adaptation and the general well-being of students.

Although exploratory, the present study identified the predictors and risk profiles of academic burnout among psychology students. The findings emphasize the importance of universities to include mental health and healthy lifestyle promotion among their university students. Intervention programs and the institution’s awareness of these issues are crucial for fostering well-adjusted students, with repercussions on the students’ well-being and academic performance.

## Figures and Tables

**Table 1:** Participants’ sociodemographic and clinical characteristics.

Participants Characteristics	*n* (%)	Mean (*SD*)	Range
Age	274	22.05 (5.82)	18–49
Gender			
Male	29 (10.6)		
Female	245 (89.4)		
Marital status			
Single or divorced	253 (92.3)		
Dating or married	21 (7.7)		
Educational level			
Undergraduate	199 (72.6)		
Graduate	75 (27.4)		
Course satisfaction			
Dissatisfied	8 (2.9)		
Neutral	19 (6.9)		
Satisfied	18 (6.6)		
Very satisfied	229 (83.6)		
Chronic illness			
Yes	62 (22.6)		
No	212 (77.4)		
Medication			
Yes	74 (27.0)		
No	200 (73.0)		
Perceived health status			
Bad	5 (1.8)		
Fair	65 (23.7)		
Good	154 (56.2)		
Very good	50 (18.2)		
Tobacco use			
Yes	36 (13.1)		
No	238 (86.9)		
Substance use			
Yes	18 (6.6)		
No	256 (93.4)		
Alcohol consumption			
Yes	165 (60.2)		
No	109 (39.8)		
Psychological support			
Yes	42 (15.3)		
No	232 (84.7)		

**Table 2:** Correlations between psychological and lifestyle variables and academic burnout.

Variables	1	2	3	4	5	6	7
1. Psychological distress	-	0.678 **	−0.749 **	0.084	0.522 **	−0.068	0.560 **
2. Difficulties in emotional regulation		-	−0.539 **	0.017	0.352 **	−0.049	0.457 **
3. Psychological well-being			-	−0.012	−0.486 **	−0.006	−0.610 **
4. Physical activity				-	0.033	−0.127 *	−0.024
5. Sleep quality					-	0.011	0.336 **
6. Dietary inflammatory index						-	0.169
7. Academic burnout							-
Mean	16.87	83.51	16.53	1.95	6.46	−0.198	2.33
*SD*	11.820	21.471	5.218	0.705	2.868	2.159	0.936

Note. *SD* = Standard deviation; * *p* < 0.05, ** *p* < 0.01.

**Table 3:** Average scores for each variable in the risk profiling.

Profile	Psychological Distress	Psychological Well-Being	Difficulties in Emotional Regulation	Sleep Quality	Physical Activity	Dietary Inflammatory Index
Burnout Risk	24.6	12.9	96.9	8.10	1.79	1.69
No Risk	14.7	17.6	79.6	6	2	−0.774

**Table 4:** Variable cutoffs per risk profile.

Profile	Psychological Distress	Psychological Well-Being	Difficulties in Emotional Regulation	Sleep Quality	Physical Activity	Dietary Inflammatory Index
	Min–Max	Min–Max	Min–Max	Min–Max	Min–Max	Min–Max
Burnout Risk	11–53	3–20	72–132	4–15	1–3	0.07–3.39
No Risk	0–50	6–29	36–137	0–13	1–3	4.20–3.56

**Table 5:** Performance metrics of three machine learning models (Random Forest, SVM, and XGBoost).

	Accuracy	Precision	Recall	F1 Score
Random Forest	95.06%	88.89%	88.89%	88.89%
SVM	93.82%	93.34%	77.78%	84.85%
XGBoost	97.53%	90.00%	99.99%	94.73%

## Data Availability

The data presented in this study are available on request from the corresponding author. The data are not publicly available due to privacy and ethical restrictions.

## Abbreviations

The following abbreviations are used in this manuscript:

DASS21	Depression, Anxiety, and Stress Scale
DERS	Difficulties in Emotion Regulation Scale
DII	Dietary Inflammatory Index
IPAQ	International Physical Activity Questionnaire—Short Version
MBI-SS	Maslach Burnout Inventory—Student Survey
PSQI	Pittsburgh Sleep Quality Index
QFA	Food Frequency Questionnaire
QGBEP	Psychological General Well-being Index
SD	Standard deviation
SEM	Structural Equation Modeling
SVM	Support Vector Machine
